# Identification of four novel serum protein biomarkers in sepsis patients encoded by target genes of sepsis-related miRNAs

**DOI:** 10.1042/CS20130301

**Published:** 2014-03-05

**Authors:** Hui-juan Wang, Bao-zeng Wang, Peng-jun Zhang, Jie Deng, Zhi-rui Zhao, Xin Zhang, Kun Xiao, Dan Feng, Yan-hong Jia, You-ning Liu, Li-xin Xie

**Affiliations:** *Department of Respiratory Medicine, Chinese PLA General Hospital, Beijing, 100853, China; †Medical College, Nankai University, Tianjin, P. R. 300071, China; ‡Department of Respiratory Medicine, the Affiliated Beijing Tiantan Hospital of Capital Medical University, Beijing, 100050, China; §Department of Clinical Biochemistry, Chinese PLA General Hospital, Beijing, 100853, China; ∥Department of Respiratory Medicine, Beijing Nanyuan Hospital, Beijing, 100076, China; ¶Department of Medical Statistics, Chinese PLA General Hospital, Beijing, 100853, China

**Keywords:** ACVR2A (activin A receptor, type IIA), FOXO1 (forkhead box O1), IHH (Indian hedgehog), miRNA, sepsis, STK4 (serine/threonine kinase 4), *ACVR2A*, activin A receptor, type IIA, AIC, Akaike information criterion, APACHE II, Acute Physiology and Chronic Health Evaluation II, AUC, area under the curve, CI, confidence interval, CRP, C-reactive protein, *DUSP3*, dual specificity phosphatase 3, *FOXO1*, forkhead box O1, *FRS2*, factor receptor substrate 2, GO, Gene Ontology, ICU, intensive care unit, *IHH*, Indian hedgehog, IL-6, interleukin 6, IL-18, interleukin 18, KEGG, Kyoto Encyclopedia of Genes and Genomes, PCT, procalcitonin, ROC, receiver operating characteristic, *SLC4A4*, solute carrier family 4, member 4, SOFA, Sequential Organ Failure Assessment, *STK4*, serine/threonine kinase 4, TGF-β, transforming growth factor-β

## Abstract

The goal of the present study was to identify novel protein biomarkers from the target genes of six serum miRNAs that we identified previously in patients with sepsis. The target genes were predicted by bioinformatics analysis; the levels of the respective proteins in the sera of patients with sepsis were detected by ELISA. *ACVR2A* (activin A receptor, type IIA), *FOXO1* (forkhead box O1), *IHH* (Indian hedgehog), *STK4* (serine/threonine kinase 4) and *DUSP3* (dual specificity phosphatase 3) were predicted to be the targets of the six miRNAs, and their encoded proteins were used for biomarker identification. Levels of ACVR2A (*P*<0.01) and FOXO1 (*P*<0.01) were significantly different among normal controls, patients with sepsis, patients with severe sepsis and patients with septic shock. Furthermore, levels of ACVR2A (*P*=0.025), FOXO1 (*P*<0.001), IHH (*P*=0.001) and STK4 (*P*=0.001) were differentially expressed in survivors and non-survivors. DUSP3 levels were not significantly different between any groups. Conjoin analysis of the four differentially expressed proteins showed that the area under the curve of the predictive probabilities was 0.875 [95% CI (confidence interval): 0.785–0.965], which was higher than the SOFA (Sequential Organ Failure Assessment) and APACHE II (Acute Physiology and Chronic Health Evaluation II) scores. When the value of predictive probabilities was 0.449, the four proteins yielded a sensitivity of 68% and a specificity of 91%. Dynamic changes in ACVR2A, FOXO1 and IHH levels showed differential expression between survivors and non-survivors at all time points. On the basis of a combined analysis of the four identified proteins, their predictive value of 28-day mortality of patients with sepsis was better than the SOFA or APACHE II scores.

## Abstract 2

Four novel protein biomarkers encoded by the miRNA target genes were identified for patients with sepsis. The combined analysis of the four proteins indicated that their predictive value for sepsis prognosis was better than the values for the SOFA score and APACHE II score.

## INTRODUCTION

Sepsis is a leading cause of death in ICUs (intensive care units). Excessive inflammatory and anti-inflammatory responses are involved in the sepsis process [1,2]; many other important biological processes are also involved [3–5]. Biomarkers of sepsis allow early intervention that can reduce the risk of death [6,7]. Many commonly used biomarkers have been identified to diagnose sepsis or evaluate sepsis severity. CRP (C-reactive protein) [8] and PCT (procalcitonin) are the most commonly used protein biomarkers for patients with sepsis. The SOFA (Sequential Organ Failure Assessment) score and APACHE II (Acute Physiology and Chronic Health Evaluation II) score are used to evaluate the severity of sepsis in patients to clinically guide treatment [9,10]. However, CRP cannot been used as a biomarker for prognosis evaluation of patients with sepsis on their first day of admission [11], and PCT has low sensitivity and specificity for predicting the mortality of sepsis [12]. Furthermore, the SOFA and APACHE II scores cannot be used as treatment targets for sepsis patients. Hence there is a need to identify new sepsis biomarkers that can aid in making therapeutic decisions and add information for the screening, diagnosis, risk stratification and monitoring of the response to therapy [13].

miRNAs are approximately 22-nt long endogenous RNAs that can play important regulatory roles in animals and plants by targeting mRNAs for cleavage or translational repression [14]. miRNAs are involved in many processes including cell death, cell proliferation, haematopoiesis and patterning of the nervous system [15]. Serum miRNAs are newly emerging biomarkers for sepsis. Indeed, in two previous studies of candidate miRNAs related to immunity, *miR-223*, *miR-146a* and *miR-15a* were shown to distinguish patients with sepsis from SIRS (systemic inflammatory response syndrome) patients [16,17]. *miR-150* was the first miRNA to be identified in a genome-wide array for sepsis patients; levels of *miR-150* were reduced in both the leucocytes and sera of patients with sepsis versus normal controls [18]. Target gene prediction of *miR-150* indicated that the gene encoding IL-18 (interleukin 18) was one of its targets, and the levels of IL-18 were negatively correlated with serum *miR-150* levels. The present study demonstrated a novel technique for biomarker discovery. We have recently identified the following six serum miRNAs that are differentially expressed between survivors and non-survivors of sepsis: *miR-223*, *miR-15a*, *miR-16*, *miR-122*, miR-193b* and *miR-483-5p* [19]. These six miRNAs can be used as predictors for mortality of patients with sepsis. Differential expression of miRNAs can affect the expression of their target genes, leading to changes in the levels of the proteins that they encode.

Hence, in the present study, we first predicted the target genes of the six miRNAs by bioinformatics analysis. Then, the levels of the proteins encoded by the target genes were determined in the sera of 125 patients with sepsis. Dynamic changes of these proteins were evaluated in another 21 patients with sepsis at six different time points.

## MATERIALS AND METHODS

### Bioinformatics analysis

In our previous study [19], we identified six miRNAs as prognostic predictors of sepsis patients. Compared with patients that survived, the levels of *miR-223*, *miR-15a* and *miR-16* were down-regulated and the levels of *miR-122*, miR-193b* and *miR-483-5p* were up-regulated in the non-surviving patient group. In the present study, the Targetscan (http://www.targetscan.org/) and miRanda (http://www.microrna.org/microrna/home.do) databases were used to identify the target genes of the six miRNAs. The common target genes identified by these two databases were used for GO (Gene Ontology) analysis and KEGG (Kyoto Encyclopedia of Genes and Genomes) analysis.

GO analysis was applied to analyse the primary function of the differentially expressed genes according to GO, which is the key functional classification of the National Center for Biotechnology Information [20–22]. Similarly, pathway analysis was used to determine the most significant pathway of the differentially expressed genes according to KEGG [23–25]. The common target genes were entered into the GO analysis website and KEGG website to obtain enriched GO terms and significant KEGG pathways. The target genes that were present in both the enriched GO terms and significant KEGG pathways were used for further analysis. miRNA–gene relationships were measured by their differential expression values, and the miRNA–gene network was built according to the interactions of miRNA and genes in the Sanger miRNA database. The centre of the network was represented by degree, which indicates the contribution of one miRNA to the genes around it or the contribution of one gene to the miRNAs around it. Key miRNAs and genes in the network have a larger degree value [26,27]. The bioinformatics analysis was performed by the Gminix company in Shanghai, China. The flow diagram is shown in Supplementary Figure S1 at http://www.clinsci.org/cs/126/cs1260857add.htm.

### Ethics

All patients and normal controls gave their written informed consent. The present study was approved by the ethics committee of the Chinese PLA General Hospital.

### Study population

**Figure 1:**
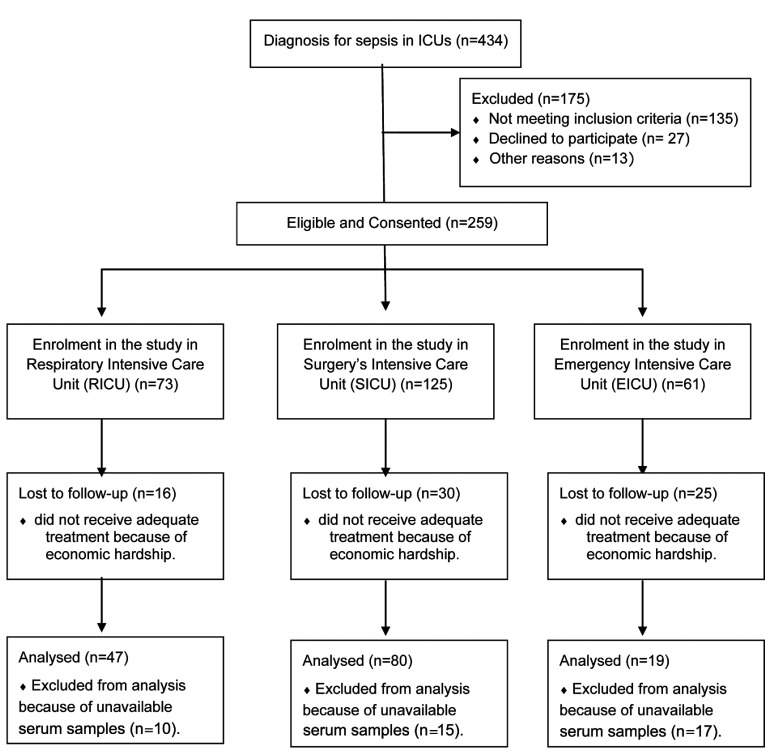
The Consort recruiting process for 146 patients with sepsis

Blood samples from 146 patients with sepsis were collected within 24 h after a positive diagnosis of sepsis. These patients were in the RICU (respiratory ICU), EICU (emergency ICU) or Department of Surgery ICU of the Chinese PLA General Hospital from July 2010 to February 2012. Of these 146 patients, 11 survivors and ten non-survivors were used in the present study for dynamic change analysis, and their blood samples were collected on days 1, 3, 5, 7, 10 and 14 after admission. The recruiting process met the Consort standard, and the Consort flow diagram is shown in Figure 1. A total of 30 healthy controls were recruited from the Health Screening Center of the Chinese PLA General Hospital for the present study. They were matched by sex and age with the sepsis patients, and they did not have any type of known infection or known medical condition.

All patients with sepsis met the criteria developed in 2003 by the American College of Chest Physicians/Society of Critical Care Medicine [28]. Patients with sepsis who progressed to at least one organ dysfunction were defined as having severe sepsis, and those patients who progressed to circulatory failure were defined as having septic shock. All patients recruited in the study were adults (≥18 years old) and fulfilled the definition for mild sepsis, severe sepsis or septic shock. Patients who had granulocyte counts of <0.5×10^9^/l, those who could not receive adequate treatment because of economic hardship, and those who received a massive transfusion or a liquid resuscitation before their admissions to ICU were excluded from the present study.

### Measurement of serum proteins

Serum levels of proteins were determined using an enzyme-linked fluorescence analysis kit (Antibody-Online). Serum CRP levels were determined by scattering turbidimetry (CardioPhase hsCRP; Siemens), and PCT levels were determined using an enzyme-linked fluorescence analysis kit (ELFA, VIDAS® BRAHMS PCT™ kit; bioMérieux). All procedures followed the manufacturers’ instructions, and duplicate measurements were performed for each sample.

### Statistical analysis

Normally distributed variables are given as means±S.D., and these values were compared between groups using the Student's *t* test. Non-normally distributed variables are summarized as medians, and the two groups were compared using the Mann–Whitney *U* test. To determine the diagnostic value of variables, ROC (receiver operating characteristic) curves were generated, and the AUC (area under the curve) was calculated. Binary logistic regression analysis was used to calculate predictive probabilities of the differentially expressed proteins. AIC (Akaike information criterion) was also used to compare the merits of the diagnostic model. Pair-wise comparison (Wilcoxon test) was used to compare the difference in protein levels between two neighbouring time points. The statistical significance was set at *P*<0.05. SPSS 20.0 software was used for all statistical analyses.

## RESULTS

### Identification of sepsis-related target genes

The miRanda website was used to search a total of 12413 predicted target genes of the six miRNAs, and a total of 2591 target genes were searched using the TargetScan website. Only 816 predicted target genes were identified as targets by both websites (the target genes identified using Targetscan and miRanda are available as Supplementary Online Data at http://www.clinsci.org/cs/126/cs1260857add.htm). The KEGG pathway and GO enrichment analyses were performed on these 816 predicted target genes. The enriched GO terms for up-regulated miRNAs in the sera of non-survivors (*miR-483-5p*, miR-193b* and *miR-122*) indicated that they are negative regulators of apoptosis (GO:0043066) and are involved in the production of siRNAs for RNA interference (GO:0030422). An additional 52 GO terms were also enriched.

Regulation of transcription, DNA-dependent cell differentiation (GO:0006355), multicellular organism development (GO:0007275) and another 23 GO terms were enriched for the target genes of the down-regulated miRNAs (*miR-16*, *miR-15a* and *miR-223*) (Supplementary Figure S2 at http://www.clinsci.org/cs/126/cs1260857add.htm). KEGG pathway analysis showed that the JAK (Janus kinase)/STAT (signal transducer and activator of transcription)/MAPK (mitogen-activated protein kinase) pathway and cancer signalling pathways were abundant pathways for target genes of the up-regulated miRNAs. The insulin, Wnt, HTLV-I (human T-lymphotropic virus type 1) infection, neurotrophin and cell cycle signalling pathways were the abundant pathways for targets of the down-regulated miRNAs (Supplementary Figure S3 at http://www.clinsci.org/cs/126/cs1260857add.htm). We then identified target genes involved in both the GO and KEGG enriched pathways (the common pathway and target genes identified after GO and KEGG analyses are available as Supplementary Online Data at http://www.clinsci.org/cs/126/cs1260857add.htm).

**Figure 2:**
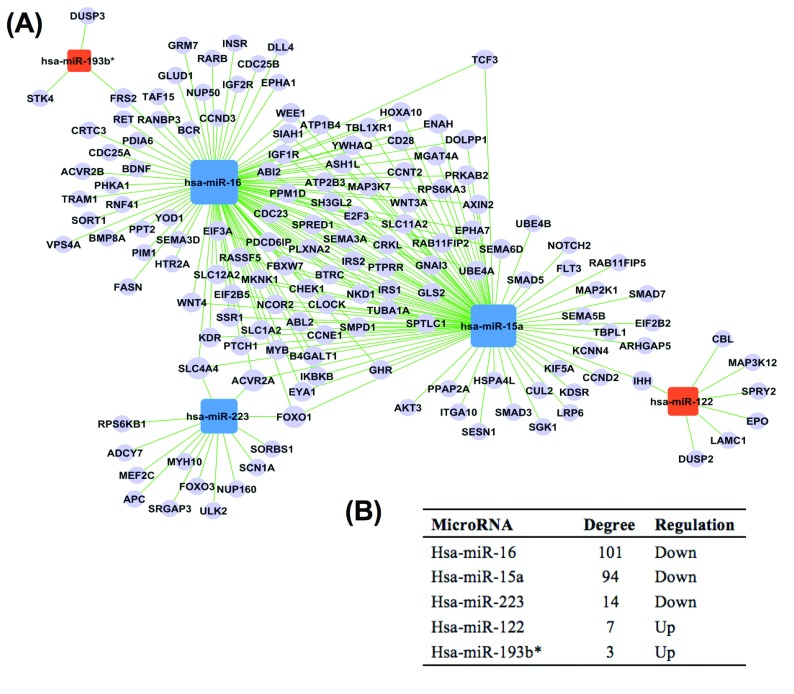
The network of miRNAs (**A**), their target genes and the degrees for each miRNA (**B**)

To analyse the interactions between the miRNAs and genes, a miRNA–gene network was built (Figure 2A). In this network, *ACVR2A* (activin A receptor, type IIA), *FOXO1* (forkhead box O1) and *SLC4A4* (solute carrier family 4, member 4) were the common target genes of *miR-223*, *miR-15a* and *miR-16* (with a degree of 3). *IHH* (Indian hedgehog) was the common target gene of *miR-122* and *miR-15a* with a degree of 2. *FRS2* (factor receptor substrate 2) was the common target gene of miR-193b* and *miR-16*. Although *STK4* (serine/threonine kinase 4) and *DUSP3* (dual specificity phosphatase 3) were only target genes of miR-193b*, these two genes were also selected because the level of miR-193b* had the highest predictive value of sepsis mortality [19] (Table 1). According to the KEGG pathway database, *SLC4A4* is only involved in pancreatic and bile secretion, and *FRS2* is only involved in the neurotrophin signalling pathway. Therefore we selected *ACVR2A*, *FOXO1*, *IHH*, *STK4* and *DUSP3* for further biomarker identification.

**Table 1:** Selected genes for further analysis

Gene	Degree	miRNAs
*ACVR2A*	3	*miR-223*, *miR-15a* and *miR-16*
*FOXO1*	3	*miR-223*, *miR-15a* and *miR-16*
*SLC4A4*	3	*miR-223*, *miR-15a* and *miR-16*
*IHH*	2	*miR-122* and *miR-15a*
*FRS2*	2	miR-193b* and *miR-16*
*DUSP3*	1	miR-193b*
*STK4*	1	miR-193b*

### Clinical data of participants

A total of 146 patients with sepsis and 30 healthy controls were included in the study. The patients with sepsis and healthy controls were matched by sex (*P*=0.523) and age (*P*=0.219). Sepsis resulted from pulmonary and abdominal infection in 54 and 28% respectively, in the sepsis patients. Of those 146 sepsis patients, 48 had mild sepsis, 66 had severe sepsis and 32 had septic shock; the overall mortality was 37% (Table 2).

**Table 2:** Clinical characteristic of the 146 patients with sepsis and 30 healthy controls The *P* values for sex and age were calculated by χ^2^ and Mann–Whitney *U* tests respectively. The values for the APACHE II score, SOFA score, CRP, PCT and WBC are medians, with the two values in parentheses being the minimum and maximum respectively. CHD, coronary heart disease; COPD, chronic obstructive pulmonary disease; WBC, white blood cell.

Variables	Healthy controls (*n*=30)	Sepsis patients (*n*=146)	*P* value
Sex (*n*) (male/female)	20/10	95/51	0.523
Age (years)	58 (50–68)	60 (20–87)	0.219
Source of infection			
Lung (*n*)	–	79 (54.1%)	–
Abdomen (*n*)	–	41 (28.1%)	–
Catheter/blood stream (*n*)	–	16 (10.9%)	–
Other (*n*)	–	10 (6.9%)	–
APACHE II score	–	20.28 (5, 38)	–
SOFA score	–	7.20 (0, 16)	–
CRP (mg/dl)	–	9.96 (0.3, 32.0)	–
PCT (ng/ml)	–	12.98 (0.05, 191.4)	–
WBC (×10^9^/l)	–	13.26 (2.5, 35.8)	–
Sepsis status			
Sepsis (*n*)	–	48 (32.88%)	–
Severe sepsis (*n*)	–	66 (45.21%)	–
Septic shock (*n*)	–	32 (21.91%)	–
Co-morbid conditions			
CHD (*n*)	–	20 (13.70%)	–
Cancer (*n*)	–	15 (10.27%)	–
Hypertension (*n*)	–	29 (19.86%)	–
Diabetes (*n*)	–	10 (6.85%)	–
COPD (*n*)	–	30 (20.55%)	–
28-day mortality (%)	–	36.99%	–

### Diagnostic value of the five proteins

The diagnostic value of the five proteins was first evaluated in the blood samples of 125 out of the 146 patients with sepsis on the first day after their admission and in the 30 healthy controls. Then, we compared the protein levels across the healthy control group, mild sepsis group, severe sepsis group and septic shock group. These data showed that levels of ACVR2A, FOXO1 and STK4 were significantly higher in the normal controls than in the patients with sepsis (*P*<0.001). The levels of ACVR2A were significantly higher in the severe sepsis group than in the mild sepsis group, and were significantly higher in the septic shock group than in the severe sepsis group. The opposite trend was observed for FOXO1; the SOFA and the APACHE II scores were at the highest levels in the mild sepsis group and at the lowest levels in the septic shock group.

**Figure 3:**
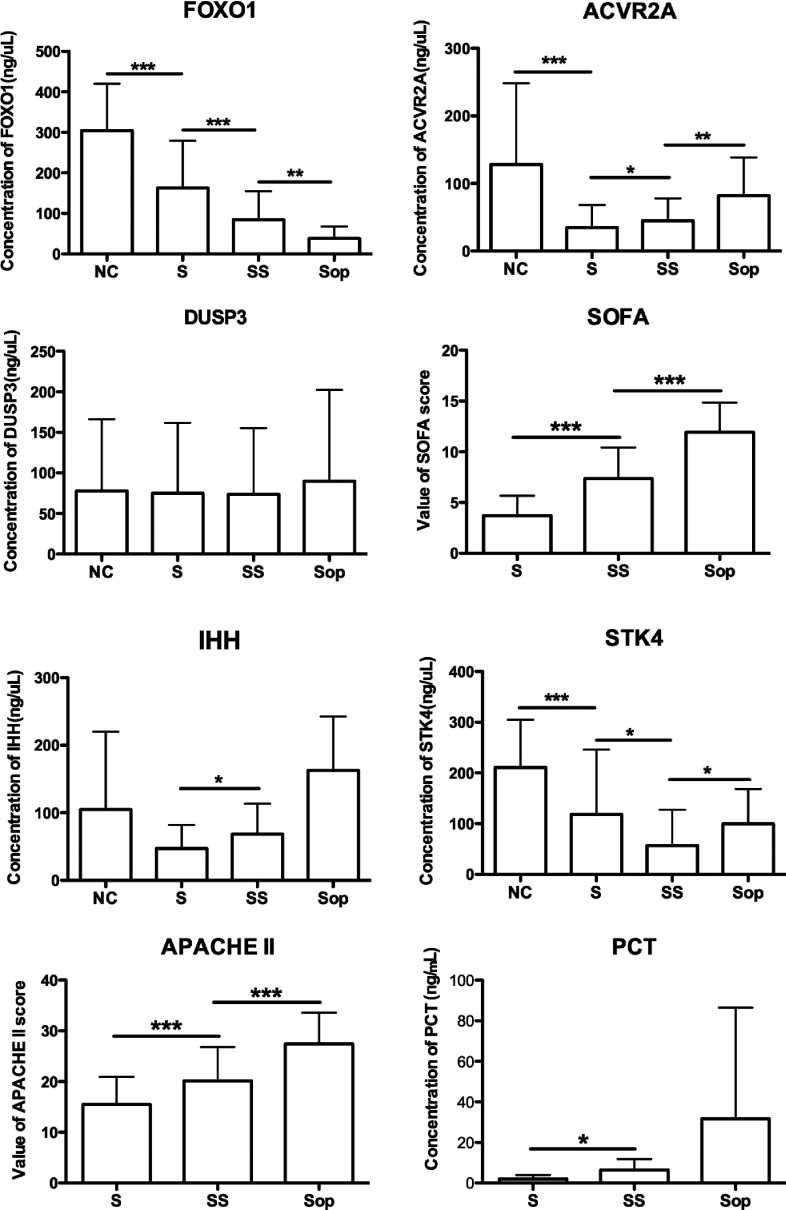
Comparison of FOXO1, ACVR2A, IHH, STK4 and DUSP3 levels, SOFA score, APACHE II score, and PCT levels Comparison of FOXO1, ACVR2A, IHH, STK4 and DUSP3 levels, SOFA score, APACHE II score, and PCT levels among normal controls (NC) (*n*=30), patients with mild sepsis (S) (*n*=42), patients with severe sepsis (SS) (*n*=56) and patients with septic shock (Sop) (*n*=27). The data are shown as means and the error bars indicate S.D. *P<0.05, **P<0.01 and ***P<0.001.

The levels of STK4 in patients with mild sepsis and patients with septic shock were both significantly higher than those in patients with severe sepsis (*P*<0.05). The levels of IHH and PCT were significantly higher in the severe sepsis group than in the mild sepsis patients; however, no significant differences in IHH or PCT levels were observed between the other groups. For DUSP3, no significant difference was observed between any two groups (Figure 3). Hence among the five proteins, only FOXO1 could be used as diagnostic biomarker for sepsis patients.

### Prognostic value of the five proteins

**Figure 4:**
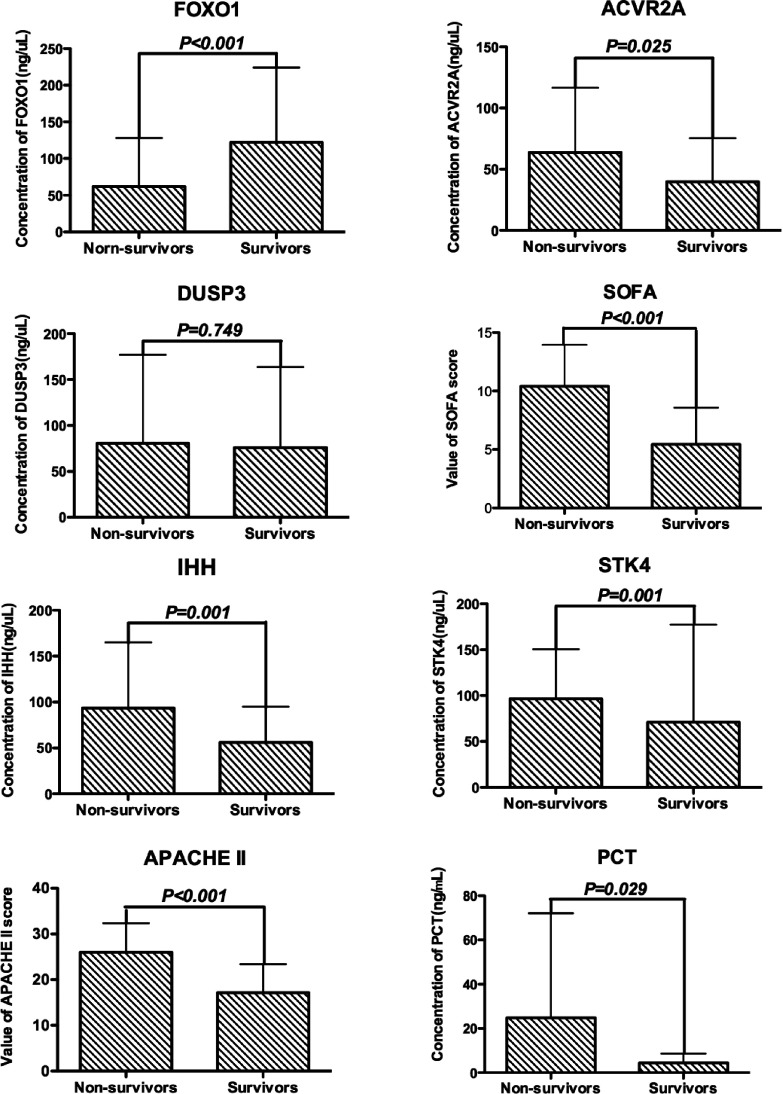
Comparison of FOXO1, ACVR2A, IHH, STK4 and DUSP3 levels, SOFA score, APACHE II score, and PCT levels between survivors (*n*=84) and non-survivors (*n*=41) The data are shown as means and the error bars indicate S.D.

To evaluate the prognostic value of the five proteins, we divided the 125 patients with sepsis into a surviving group and a non-surviving group according to 28-day mortality. The levels of FOXO1, ACVR2A, IHH and STK4 were all differentially expressed between survivors and non-survivors with *P*<0.001, *P*<0.025, *P*<0.001 and *P*<0.001 respectively, whereas the levels of DUSP3 were not differentially expressed. The SOFA score, APACHE II score and PCT were also significantly different between survivors and non-survivors with *P*<0.001, *P*<0.001 and *P*<0.029 respectively (Figure 4).

**Figure 5:**
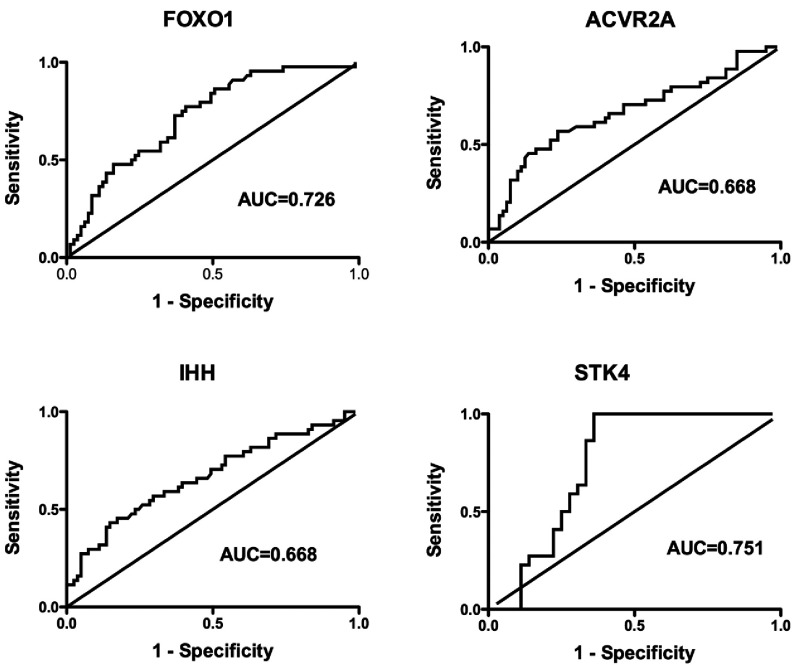
ROC curves for FOXO1, ACVR2A, IHH, and STK4 levels between survivors (*n*=84) and non-survivors (*n*=41)

**Figure 6:**
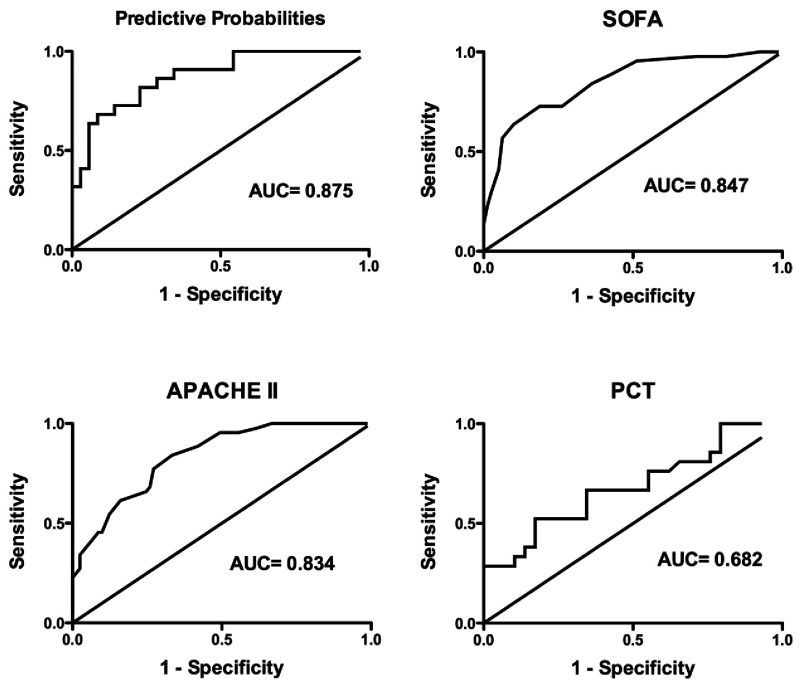
ROC curves for predictive probabilities, SOFA score, APACHE II score and PCT levels between survivors (*n*=84) and non-survivors (*n*=41)

ROC analysis was then performed on ACVR2A, FOXO1, IHH and STK4 to evaluate the predictive value of sepsis mortality. STK4 had the highest AUC of 0.751 (95% CI: 0.624–0.878) followed by FOXO1, IHH and ACVR2A with AUCs of 0.726 (95% CI: 0.636–0.816), 0.668 (95% CI: 0.566–0.770) and 0.668 (95% CI: 0.564–0.772) respectively (Figure 5) (CI stands for confidence interval). Since these four proteins originated from one miRNA profile, we entered them into a binary logistic regression analysis to obtain predictive probabilities. ROC analysis was subsequently performed on the predictive probabilities; the AUC was 0.875 (95% CI: 0.785–0.965), which was higher than the SOFA score, APACHE II score and PCT, which had AUCs of 0.847 (95% CI: 0.776–0.918), 0.834 (95% CI: 0.767–0.905) and 0.682 (95% CI: 0.529–0.835) respectively (Figure 6). When the value of the predictive probabilities was 0.449, the four proteins yielded a sensitivity of 68 and a specificity of 91%. Then, the AIC value was also calculated for the four proteins, SOFA score, APACHE II score and PCT. The AIC values were −331, −234.75, −228.25 and −300.75 respectively. The four proteins have the lowest AIC value, which meant that the four proteins had better prognostic value than other values.

### Dynamic changes of proteins in sepsis patients

**Figure 7:**
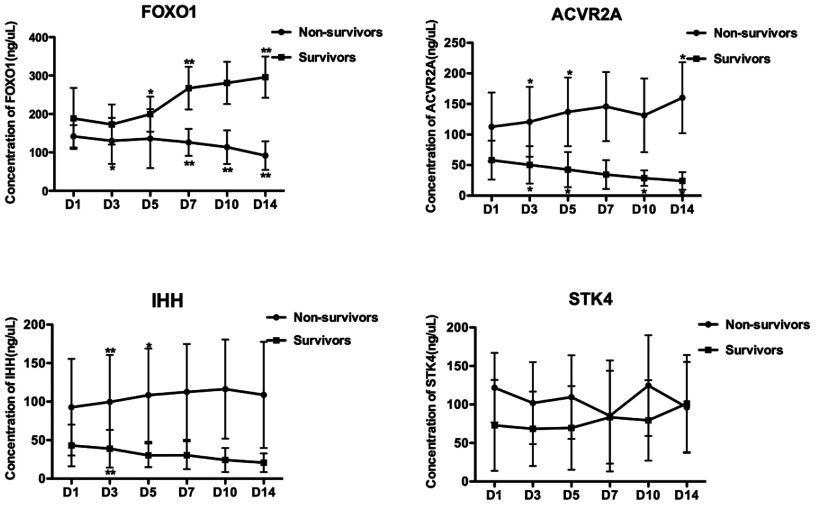
Dynamic changes of serum FOXO1, ACVR2A, IHH and STK4 levels Dynamic changes of serum FOXO1, ACVR2A, IHH and STK4 levels on day 1 (D1), day 3 (D3), day 5 (D5), day 7 (D7), day 10 (D10) and day 14 (D14) after admission in survivors (*n*=11) and non-survivors (*n*=10). The data are shown as means and the error bars indicate S.D. *P<0.05 and **P<0.01.

Dynamic changes in serum ACVR2A, FOXO1, IHH and STK4 levels in patients with sepsis during their hospitalization in ICUs were evaluated. The 21 patients with sepsis were separated into a group of ten non-survivors (two with mild sepsis, four with severe sepsis and four with septic shock) and a group of 11 survivors (four with mild sepsis, five with severe sepsis and two with septic shock) according to 28-day mortality. As shown in Figure 7, the serum levels of FOXO1, ACVR2A and IHH in the surviving group were significantly higher than those in the non-surviving group at all time points (*P*<0.001). The levels of FOXO1 in the non-surviving group showed a significantly decreasing trend over time, whereas the expression levels of FOXO1 in the surviving group tended to significantly increase during hospitalization in the ICU (*P*<0.05). Unlike FOXO1, ACVR2A showed a reversed tendency over time, with a significantly increasing trend in the non-surviving group and a significantly decreasing trend in the surviving group (*P*<0.05). For STK4, except for the first three time points, no significant difference was present between the survivors and non-survivors.

## DISCUSSION

Early diagnosis and timely treatment are crucial for improving the prognosis of patients with sepsis. The value of biomarkers for sepsis has been well established in previous studies. In the present study, one important finding was the identification of a protein expression profile to predict the prognosis of patients with sepsis. Clinically, the levels of PCT and the APACHE II and SOFA scores are used as biomarkers to evaluate the prognosis of patients with sepsis. In the present study, the PCT levels and the APACHE II and SOFA scores were also evaluated. The prognostic value of the four proteins identified in this study was better than that of the PCT levels or the APACHE II and SOFA scores. Another important finding of the present study was that FOXO1 had diagnostic value among patients with severe sepsis and septic shock. Dynamic change analysis of these proteins also showed that the levels of ACVR2A and IHH gradually increased over time in non-survivors and decreased in survivors, whereas levels of FOXO1 showed the reverse trend in these two sepsis groups. These data indicate that these proteins might be effective therapeutic targets for sepsis treatment.

In our previous study, the levels of *miR-223* [29], *miR-15a* and *miR-16* [17] were found to be higher in sepsis groups than in normal controls. This indicates that the levels of ACVR2A and FOXO1 were lower in patients with sepsis than in normal controls because they are the common target genes of *miR-223*, *miR-15a* and *miR-16*. The six miRNAs biomarkers identified in our previous study were differentially expressed between survivors and non-survivors and these survivors and non-survivors were matched by sepsis severity. Therefore these miRNAs were not related to the sepsis severity. It has also been demonstrated that miRNA levels in sera of patients with sepsis did not change over time (H.-j. Wang, B.-z. Wang, P.-j. Zhang, J. Deng, Z.-r. Zhao, X. Zhang, K. Xiao, D. Feng, Y.-h. Jia, Y.-n. Liu and L.-x. Xie, unpublished work). Hence four of the five proteins levels were significantly different between survivors and non-survivors and not related to the sepsis severity. Levels of FOXO1 and ACVR2A were differentially expressed among patients with sepsis, severe sepsis and septic shock, and the reason might be that some of these patients suffered from acute or chronic liver or kidney failure.

The *AVCR2A* gene is a Th17-specific gene, and TGF-β (transforming growth factor-β) and IL-6 (interleukin 6) are required for the induction of ACVR2A [30]. Several studies have demonstrated that serum IL-6 levels are a diagnostic and prognostic biomarker for sepsis patients [31–33]. In patients with septic shock, ARDS (acute respiratory distress syndrome) patients with a fatal outcome had higher TGF-β1 (transforming growth factor-β1) concentrations than survivors [34]. In mice with sepsis, neutralizing IL-10 (interleukin 10) or TGF-β might represent a novel strategy for treating the immunosuppressive condition associated with sepsis [35]. Hence ACVR2A may affect sepsis processes by regulating the levels of IL-6 or TGF-β.

Two metabolic characteristics of sepsis are muscle insulin resistance [36] and severe muscle wasting [37]. A previous study has provided evidence that the Akt/FOXO1 signalling pathway might be involved in this process [38]. The study by Crossland et al. [39] showed that LPS (lipopolysaccharide) infusion induced an increase in the *Akt* and *FOXO1* gene transcription levels and a decrease in the Akt and FOXO1 protein phosphorylation in a rat model, but there was no change in overall protein levels. Another study demonstrated that in HCT116 colorectal cancer cells, HeLa cervical cancer cells and HuH-7 hepatoma cells, *miR-223* regulated FOXO1 expression and cell proliferation [40]. However, these studies did not analyse blood samples; the levels of FOXO1 in sera of patients with sepsis were still lower than levels in the sera of normal controls, and the mechanism remains unclear. To date, no functional studies addressing the role of IHH and STK4 in sepsis have been reported. However, STK4 deficiency is a novel human primary immunodeficiency syndrome [41]. These protein biomarkers were first reported in patients with sepsis.

The present study has several novel aspects. First, candidate protein selection was based on the target genes of miRNAs that were identified as biomarkers for patients with sepsis. Four of five candidate proteins were confirmed as prognostic predictors, indicating that this is an effective method for protein biomarker screening. In contrast with proteomics-based screening using peptide fragments, the proteins obtained using our approach were from the coding genes, which is a more accurate approach. In addition, by using our approach, the miRNAs and the protein encoded by the target gene can both be used as biomarkers for patients with sepsis. However, both approaches use a genome-wide screen of protein biomarkers. Secondly, FOXO1 can be used as a diagnostic biomarker for patients with sepsis and can be a predictor of the prognosis of patients with sepsis. Both are novel biomarkers and are involved in the pathological process of sepsis.

However, there are a few limitations in the present study. The major limitation is that the regulatory mechanism of miRNA is intracellular; the number of proteins secreted into circulation and the regulatory mechanism of secretion are unknown. Hence the serum levels of these proteins may not correlate with the intracellular levels. As the next step of our study, confirmation of the target genes will be performed in a cell line and levels of miRNAs and their target gene-coding proteins inside the cells and culture medium will be evaluated. Additionally, we only recruited patients with sepsis from one hospital; patients with sepsis from other hospitals and other races need to be included in future studies. Thirdly, the healthy controls included in the present study were only used to provide a normal baseline for comparison. Some ICU patients who did not have sepsis should be recruited as controls for sepsis diagnosis, which will be the next step of our study.

## CLINICAL PERSPECTIVES

• Sepsis is a leading cause of death in intensive care units and there is a need to identify new biomarkers that can aid in early diagnosis and timely treatment. Six miRNAs identified in our previous study can be used as predictors for sepsis prognosis, but their target genes are unknown.
• Four novel protein biomarkers encoded by the miRNA target genes were identified for patients with sepsis. The combined analysis of the four proteins indicated that their predictive value for sepsis prognosis was better than the values for the SOFA score and APACHE II score.
• Therefore these findings indicate that the proteins identified might be suitable for diagnostic purposes and/or effective therapeutic targets for sepsis treatment.

## Online data

Supplementary data

Common target genes from the Targetscan and miRanda databases

Common target genes after GO and KEGG
