# Supplementary material for: Identification of four novel serum protein biomarkers in sepsis patients encoded by target genes of sepsis-related miRNAs
Source: Clin Sci (Lond). 2014 Mar 5;126(Pt 12):857–67. doi: 10.1042/CS20130301 (PMC4202716; doi:10.1042/CS20130301)
Supplement: Supplementary data [file cs1260857add.pdf]

Clinical Science (2014) **126**, 857–867 (Printed in Great Britain) doi: 10.1042/CS20130301

online data

Original Paper

SUPPLEMENTARY ONLINE DATA

Identification of four novel serum protein biomarkers in sepsis patients encoded by target genes of sepsis-related miRNAs

Hui-juan WANG*†[1], Bao-zeng WANG‡[1], Peng-jun ZHANG§[1], Jie DENG*‖, Zhi-rui ZHAO*, Xin ZHANG*, Kun XIAO*, Dan FENG¶, Yan-hong JIA*, You-ning LIU* and Li-xin XIE*

*Department of Respiratory Medicine, Chinese PLA General Hospital, Beijing, 100853, China
†Medical College, Nankai University, Tianjin, P. R. 300071, China
‡Department of Respiratory Medicine, the Affiliated Beijing Tiantan Hospital of Capital Medical University, Beijing, 100050, China
§Department of Clinical Biochemistry, Chinese PLA General Hospital, Beijing, 100853, China
‖Department of Respiratory Medicine, Beijing Nanyuan Hospital, Beijing, 100076, China
¶Department of Medical Statistics, Chinese PLA General Hospital, Beijing, 100853, China

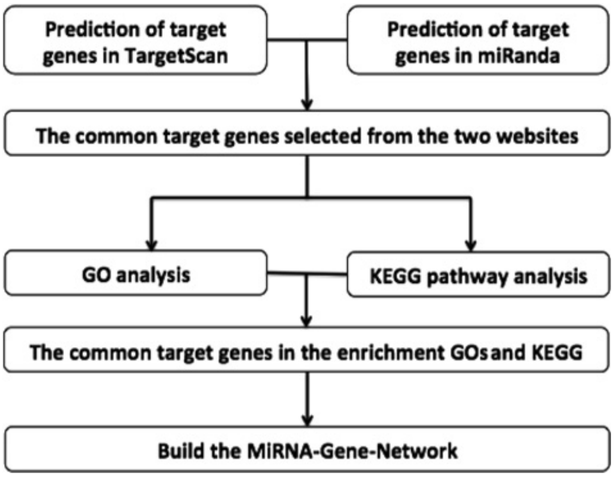

Figure S1 The flow diagram of the bioinformatics analysis

Clinical Science www.clinsci.org

[1]These authors contributed equally to this work.

Correspondence: Dr You-ning Liu or Dr Li-xin Xie (email liuyn301@126.com or xielx@263.net).

© 2014 The Author(s)
The author(s) has paid for this article to be freely available under the terms of the Creative Commons Attribution Licence (CC-BY) (http://creativecommons.org/licenses/by/3.0/) which permits unrestricted use, distribution and reproduction in any medium, provided the original work is properly cited.

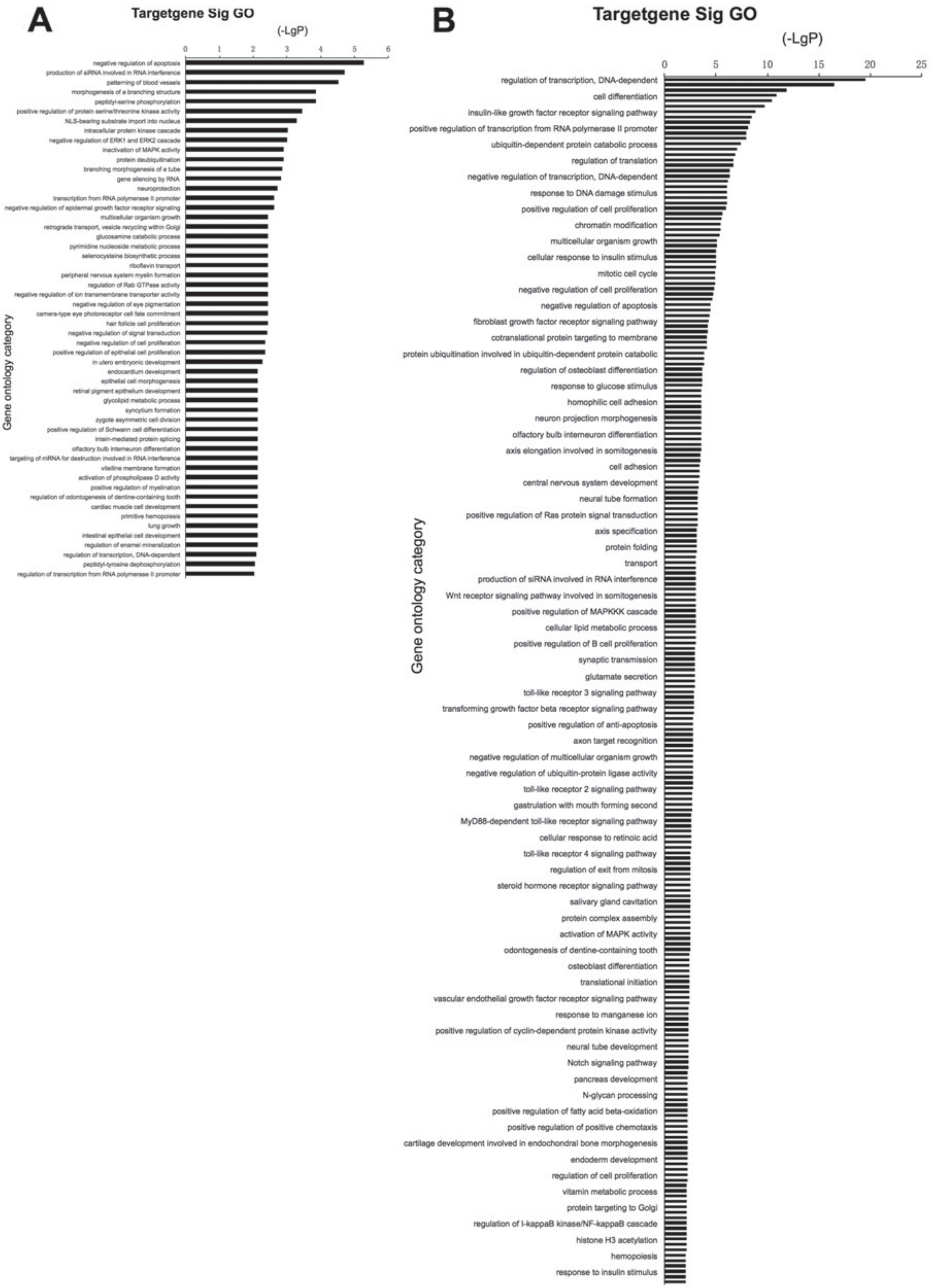

Figure S2 Significant GO terms of the target genes
(**A**) GO terms for the targets of down-regulated miRNAs. (**B**) GO terms for the targets of up-regulated miRNAs.

© 2014 The Author(s)
The author(s) has paid for this article to be freely available under the terms of the Creative Commons Attribution Licence (CC-BY) (http://creativecommons.org/licenses/by/3.0/) which permits unrestricted use, distribution and reproduction in any medium, provided the original work is properly cited.

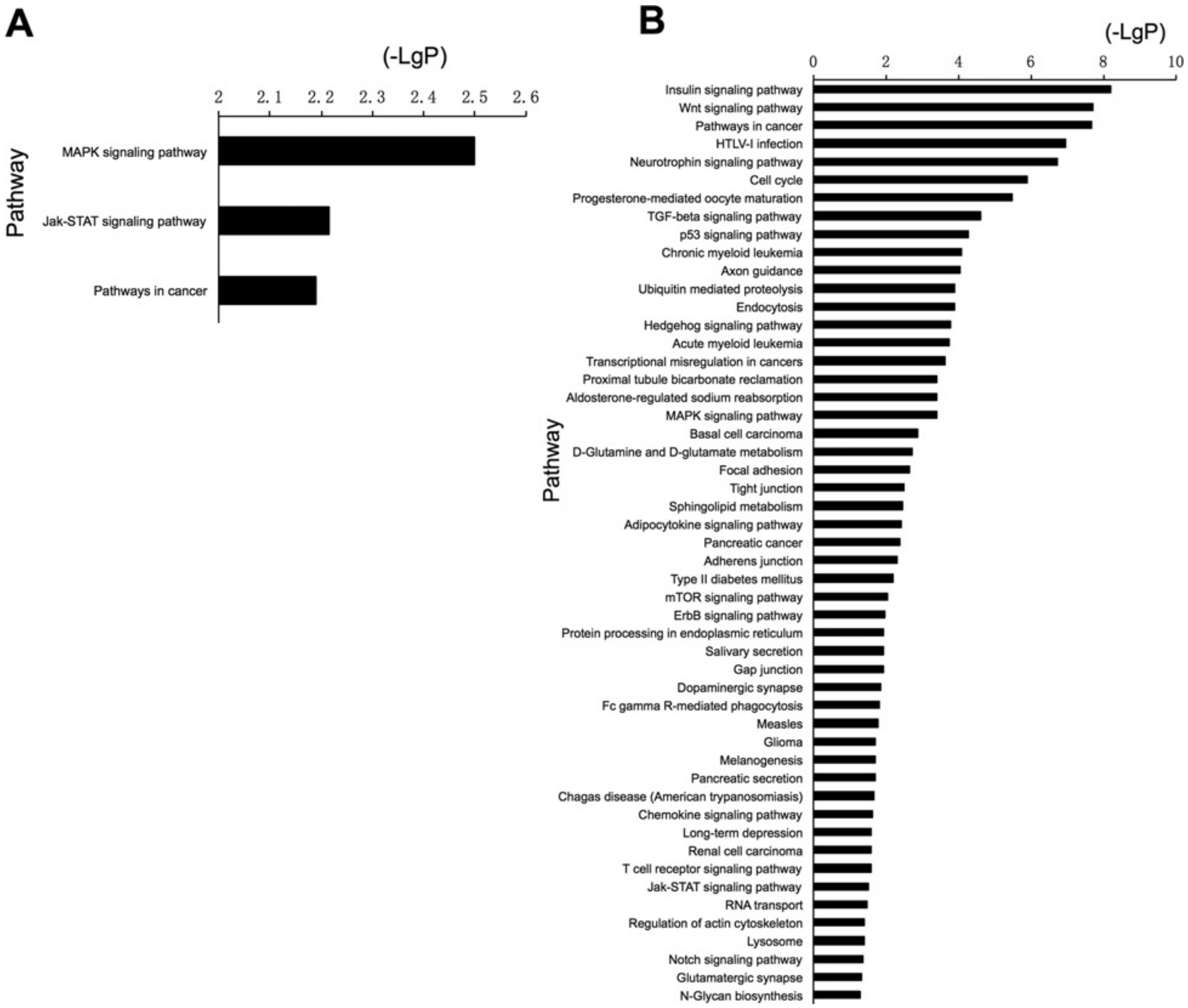

Figure S3 **Significant pathways of the target genes**
(**A**) Pathways for the targets of down-regulated miRNAs. (**B**) Pathways for the targets of up-regulated miRNAs.

Received 21 June 2013/29 October 2013; accepted 5 December 2013
Published as Immediate Publication 5 December 2013, doi: 10.1042/CS20130301

© 2014 The Author(s)
The author(s) has paid for this article to be freely available under the terms of the Creative Commons Attribution Licence (CC-BY) (http://creativecommons.org/licenses/by/3.0/) which permits unrestricted use, distribution and reproduction in any medium, provided the original work is properly cited.
